# Supplementary material for: COVID-19 vaccines effectiveness against symptomatic SARS-CoV-2 during Delta variant surge: a preliminary assessment from a case-control study in St. Petersburg, Russia
Source: BMC Public Health. 2022 Sep 22;22:1803. doi: 10.1186/s12889-022-14202-9 (PMC9503256; doi:10.1186/s12889-022-14202-9)
Supplement: Supplementary file 1 — Additional file 1: Supplementary material with the results of sensitivity analysis. [file 12889_2022_14202_MOESM1_ESM.pdf]

Supplementary materials

COVID-19 vaccines effectiveness against symptomatic SARS-CoV-2 Delta variant infection: a population-based case-control study in St. Petersburg, Russia

Anton Barchuk, Anna Bulina, Mikhail Cherkashin, Natalia Berezina, Tatyana Rakova, Darya Kuplevatskaya, Oksana Stanevich, Dmitriy Skougarevskiy, Artemiy Okhotin

Table A1. Characteristics of Cases and Controls by Vaccination Status. Sensitivity Analysis

		Overall (%)	Cases (%)	Controls (%)
	Missing vaccine names were assumed to be Gam-COVID-Vac			
Vaccine name				
	Gam-COVID-Vac (2-dose Sputnik V)	1,220 (30.9)	283 (23.6)	937 (34.1)
	Gam-COVID-Vac (1-dose Sputnik Light)	274 (6.9)	60 (5.0)	214 (7.8)
	EpiVacCorona	32 (0.8)	17 (1.4)	15 (0.5)
	CoviVac	124 (3.1)	36 (3.0)	88 (3.2)
	Other	29 (0.7)	0 (0.0)	29 (1.1)
	No vaccine	2,266 (57.4)	802 (66.9)	1,464 (53.3)
Vaccination status				
	Non-vaccinated	2,290 (58.0)	802 (66.9)	1,488 (54.2)
	Partial vaccination	59 (1.5)	13 (1.1)	46 (1.7)
	Complete vaccination	1,596 (40.5)	383 (32.0)	1,213 (44.2)
	Missing vaccine dates were assigned non-vaccination status			
Vaccine name				
	Gam-COVID-Vac (2-dose Sputnik V)	1,205 (30.5)	268 (22.4)	937 (34.1)
	Gam-COVID-Vac (1-dose Sputnik Light)	268 (6.8)	54 (4.5)	214 (7.8)
	EpiVacCorona	32 (0.8)	17 (1.4)	15 (0.5)
	CoviVac	124 (3.1)	36 (3.0)	88 (3.2)
	Other	50 (1.3)	21 (1.8)	29 (1.1)
	No vaccine	2,266 (57.4)	802 (66.9)	1,464 (53.3)
Vaccination status				
	Non-vaccinated	2311 (58.6)	803 (67.0)	1508 (54.9)
	Partial vaccination	65 (1.6)	19 (1.6)	46 (1.7)
	Complete vaccination	1,569 (39.8)	376 (31.4)	1,193 (43.4)

Table A2. Effectiveness of Vaccination against Symptomatic PCR-confirmed SARS-CoV-2. Sensitivity Analysis

		Crude OR (95% CI)	Crude VE (95% CI)	OR adjusted for age and gender (95% CI)	VE adjusted for age and gender (95% CI)
		Missing vaccine names were assumed to be Gam-COVID-Vac			
Without correcting for the history of confirmed COVID-19					
	Gam-COVID-Vac (2-dose Sputnik V)	0.58 (0.49–0.68)	42% (32–51)	0.52 (0.45–0.62)	48% (38–55)
	Gam-COVID-Vac (1-dose Sputnik Light)	0.58 (0.42–0.78)	42% (22–58)	0.55 (0.41–0.75)	45% (25–59)
	EpiVacCorona	1.86 (0.88–3.91)	-86% (-291–12)	1.64 (0.81–3.31)	-64% (-231–19)
	CoviVac	0.68 (0.44–1.06)	32% (-6–56)	0.67 (0.42–1.06)	33% (-6–58)
	Any vaccine: partial vaccination	0.52 (0.28–0.98)	48% (2–72)	0.51 (0.27–0.97)	49% (3–73)
After correcting for the history of confirmed COVID-19					
	Gam-COVID-Vac (2-dose Sputnik V)	0.50 (0.43–0.59)	50% (41–57)	0.45 (0.38–0.53)	55% (47–62)
	Gam-COVID-Vac (1-dose Sputnik Light)	0.59 (0.43–0.81)	41% (19–57)	0.56 (0.40–0.77)	44% (23–60)
	EpiVacCorona	1.60 (0.74–3.49)	-60% (-249–26)	1.40 (0.68–2.92)	-40% (-192–32)
	CoviVac	0.64 (0.41–1.01)	36% (-1–59)	0.62 (0.39–1.00)	38% (0–61)
	Any vaccine: partial vaccination	0.51 (0.27–0.97)	49% (3–73)	0.49 (0.25–0.96)	51% (4–75)
		Missing vaccine dates were assigned non-vaccination status			
Without correcting for the history of confirmed COVID-19					
	Gam-COVID-Vac (2-dose Sputnik V)	0.56 (0.47–0.65)	44% (35–53)	0.51 (0.43–0.60)	49% (40–57)
	Gam-COVID-Vac (1-dose Sputnik Light)	0.53 (0.39–0.73)	47% (27–61)	0.51 (0.37–0.70)	49% (30–63)
	EpiVacVorona	1.88 (0.89–3.96)	-88% (-296–11)	1.66 (0.82–3.34)	-66% (-234–18)
	CoviVac	0.73 (0.47–1.14)	27% (-14–53)	0.72 (0.45–1.13)	28% (-234–55)
	Other	1.22 (0.64–2.36)	-22% (-136–36)	1.26 (0.68–2.33)	-26% (-133–32)
	Any vaccine: partial vaccination	0.78 (0.45–1.33)	22% (-33–55)	0.76 (0.43–1.33)	24% (-33–57)
After correcting for the history of confirmed COVID-19					
	Gam-COVID-Vac (2-dose Sputnik V)	0.49 (0.41–0.57)	51% (43–59)	0.43 (0.37–0.51)	57% (49–63)
	Gam-COVID-Vac (1-dose Sputnik Light)	0.55 (0.39–0.76)	45% (24–61)	0.52 (0.37–0.73)	48% (27–63)
	EpiVacCorona	1.63 (0.75–3.54)	-63% (-254–25)	1.42 (0.69–2.96)	-42% (-196–31)
	CoviVac	0.70 (0.44–1.11)	30% (-11–56)	0.67 (0.42–1.09)	33% (-9–58)
	Other	1.00 (0.51–1.95)	0% (-95–49)	1.02 (0.54–1.90)	-2% (-90–46)
	Any vaccine: partial vaccination	0.76 (0.43–1.33)	24% (-33–57)	0.74 (0.41–1.33)	26% (-33–59)

Table A3. Effectiveness of Vaccination against Lung Injury. Sensitivity Analysis

		Crude OR (95% CI)	Crude VE (95% CI)	OR adjusted for age and gender (95% CI)	VE adjusted for age and gender (95% CI)
		Missing vaccine names were assumed to be Gam-COVID-Vac			
Without correcting for the history of confirmed COVID-19					
	Gam-COVID-Vac (2-dose Sputnik V)	0.44 (0.37–0.54)	56% (46–63)	0.37 (0.31–0.45)	63% (55–69)
	Gam-COVID-Vac (1-dose Sputnik Light)	0.49 (0.34–0.71)	51% (29–66)	0.48 (0.33–0.69)	52% (31–67)
	EpiVacCorona	1.58 (0.72–3.43)	-58% (-243–28)	1.27 (0.62–2.57)	-27% (-157–38)
	CoviVac	0.63 (0.38–1.05)	37% (-5–62)	0.62 (0.37–1.04)	38% (4–63)
	Any vaccine: partial vaccination	0.51 (0.25–1.04)	49% (-4–75)	0.49 (0.23–1.03)	51% (-3–77)
After correcting for the history of confirmed COVID-19					
	Gam-COVID-Vac (2-dose Sputnik V)	0.39 (0.32–0.48)	61% (52–68)	0.32 (0.26–0.39)	68% (61–74)
	Gam-COVID-Vac (1-dose Sputnik Light)	0.50 (0.34–0.72)	50% (28–66)	0.47 (0.32–0.70)	53% (30–68)
	EpiVacCorona	1.37 (0.62–3.03)	-37% (-203–38)	1.08 (0.52–2.22)	-8% (-122–48)
	CoviVac	0.60 (0.36–1.01)	40% (-1–64)	0.57 (0.33–0.98)	43% (2–67)
	Any vaccine: partial vaccination	0.50 (0.24–1.05)	50% (-5–76)	0.49 (0.22–1.06)	51% (-6–78)
		Missing vaccine dates were assigned non-vaccination status			
Without correcting for the history of confirmed COVID-19					
	Gam-COVID-Vac (2-dose Sputnik V)	0.42 (0.35–0.51)	58% (49–65)	0.35 (0.29–0.43)	65% (57–71)
	Gam-COVID-Vac (1-dose Sputnik Light)	0.46 (0.31–0.67)	54% (33–69)	0.44 (0.30–0.65)	56% (35–70)
	EpiVacVorona	1.59 (0.73–3.47)	-59% (-247–27)	1.28 (0.63–2.60)	-28% (-160–37)
	CoviVac	0.67 (0.40–1.12)	33% (-12–60)	0.65 (0.39–1.10)	35% (-10–61)
	Other	1.17 (0.58–2.37)	-17% (-137–42)	1.17 (0.60–2.27)	-17% (-127–40)
	Any vaccine: partial vaccination	0.72 (0.39–1.32)	28% (-32–61)	0.69 (0.36–1.32)	31% (-32–64)
After correcting for the history of confirmed COVID-19					
	Gam-COVID-Vac (2-dose Sputnik V)	0.37 (0.31–0.46)	63% (54–69)	0.31 (0.25–0.38)	69% (62–75)
	Gam-COVID-Vac (1-dose Sputnik Light)	0.46 (0.31–0.69)	54% (31–69)	0.44 (0.29–0.66)	56% (34–71)
	Epivaccorona	1.38 (0.62–3.06)	-38% (-206–38)	1.09 (0.53–2.25)	-9% (-125–47)
	CoviVac	0.65 (0.38–1.09)	35% (-9–62)	0.61 (0.36–1.05)	39% (-5–64)
	Other	0.97 (0.48–1.99)	3% (-99–52)	0.95 (0.48–1.88)	5% (-88–52)
	Any vaccine: partial vaccination	0.70 (0.37–1.32)	30% (-32–63)	0.68 (0.35–1.34)	32% (-34–65)
